# Impact of hyperchloremia on inflammatory markers, serum creatinine, hemoglobin, and outcome in critically ill patients with COVID-19 infection

**DOI:** 10.25122/jml-2023-0013

**Published:** 2023-05

**Authors:** Shaya Yaanallah Al Qahtani

**Affiliations:** 1. Department of Internal Medicine and Critical Care, College of Medicine, Imam Abdulrahman bin Faisal University, Dammam, Saudi Arabia

**Keywords:** Hyperchloremia, COVID-19, inflammatory markers, ferritin, CRP, critical care, AKI – Acute Kidney Injury, COVID-19 – Coronavirus Disease 2019, CRP – C-Reactive Protein, LDH – Lactate Dehydrogenase, RRT – Renal Replacement Therapy

## Abstract

Hyperchloremia has negative consequences, such as increased proinflammatory mediators, renal dysfunction, and mortality in patients with septic shock. However, data on the effects of hyperchloremia on COVID-19 infections are scarce. The study aimed to investigate the effects of hyperchloremia on inflammatory markers, serum creatinine, hemoglobin levels, and outcomes in critically ill COVID-19 patients. A retrospective review of all adult patients admitted to the ICU at King Fahd University Hospital with a moderate to severe COVID-19 infection from January 2020 to August 2021 was performed. Serum chloride levels, ferritin, lactate dehydrogenase (LDH), C-reactive protein (CRP), creatinine, and hemoglobin levels were collected on the first and third days of ICU admission. Demographic data, oxygen support modality, ICU length of stay (ICU LOS), renal replacement therapy (RRT), and deaths were collected. Of 420 patients, 255 were included; 97 (38%) had hyperchloremia, while 158 (62%) did not. Hyperchloremic patients had a higher percentage of increases in ferritin (54.6%), CRP (6.2%), and LDH (15.5%) between the first and third days of admission, compared to non-hyperchloremic patients (43.7%, 6.3%, and 5.7%, respectively). The decrease in hemoglobin levels was similar in both groups (p=0.103). There was a significant association between hyperchloremia and an increase in serum creatinine (p<0.0001). Sixty-six (68%) patients required endotracheal intubation in the hyperchloremic group (p=0.003). The mortality rate was significant in the hyperchloremic cohort (p=<0.0001). Hyperchloremia was significantly associated with increased risks of kidney injury, endotracheal intubation, and death. However, hyperchloremia was not associated with increased ferritin, CRP, or hemoglobin decreases in critically ill COVID-19 patients.

## INTRODUCTION

Severe COVID-19 is characterized by an intense inflammatory response and massive cytokine production, resulting in organ dysfunction [1]. The exact pathogenesis of COVID-19 remains poorly understood. However, multiple previous studies have proposed mechanisms by which severe acute respiratory syndrome coronavirus-2 (SARS-CoV-2) infection causes hyperinflammation and organ damage. The most prominent mechanism is the ability of the SARS-CoV-2 spike protein to bind to the angiotensin-converting enzyme 2 (ACE2) receptor [1]. The S protein has been confirmed to be the primary protein that mediates the binding of SARS-CoV-2 to the ACE2 receptor of the host cell and facilitates viral entry into cells [1]. The concentration of the COVID-19 virus is highest in tissues that contain ACE2 receptors, such as the epithelial cells found in the tracheobronchial tree, type 2 pneumocytes, endothelial cells, and cardiomyocytes [2]. The susceptibility of these specific cells may explain the various symptoms that are commonly observed among COVID-19 patients. The interaction between SARS-CoV-2 and the ACE2 receptor leads to the downregulation of protective ACE2, resulting in a dysregulated inflammatory response and oxidative stress [3]. The effect of the dysregulated inflammatory response can result in immune-mediated tissue damage and organ dysfunction. Therefore, the optimal objective is to maintain a balance in the immune response, which may eliminate the virus and prevent inflammation-mediated tissue injury [1].

Serum chloride is considered the most abundant anion in the extracellular fluid, contributing to almost one-third of extracellular fluid osmolality [4]. Serum chloride plays a significant role in maintaining body physiology and hemostasis, including acid-base balance, immune modulation, muscle contractility, and serum osmolality [5]. Moreover, chloride is essential for gastric motility, maintaining coagulation, and cardiac contractility [6]. Dyschloremia is commonly observed in critically ill patients after fluid resuscitation or diuretic therapy. Hyperchloremia is more common in patients requiring intensive care unit (ICU) admission than hypochloremia. In the setting of hyperchloremia, kidney perfusion can be affected by renal vasoconstriction, which leads to acute kidney injury (AKI) and suppression of erythropoietin, resulting in anemia [6].

Moreover, hyperchloremic acidosis can significantly augment inflammatory mediators, including interleukin-10 (IL-10) and IL-6, through activation of the nuclear factor kappa B (NF-kB) pathway [7]. Activation of the NF-kB pathway can increase inflammatory markers such as C-reactive protein (CRP) [8]. The relationship between inflammatory markers and the severity of COVID-19 infections has been extensively reviewed in previous literature. Elevated levels of inflammatory markers, namely, CRP, lactate dehydrogenase (LDH), ferritin, and D-dimer, in COVID-19 infections are associated with disease severity and poor outcomes [9,10]. Furthermore, patients with COVID-19 who exhibit elevated CRP levels upon admission are more likely to experience hypoxia and respiratory failure than others [11]. The deleterious effects of hyperchloremia on the prognosis and outcomes of septic patients have been reviewed in previous literature. Hyperchloremia can result in increased morbidity and death in non-COVID-19 septic shock patients [12,13]. However, data on the effects of hyperchloremia on COVID-19 infections are scarce. As a result, we hypothesized that hyperchloremia could exacerbate the inflammatory response and negatively impact outcomes in COVID-19 patients.

This study aimed to describe the impact of hyperchloremia on inflammatory markers, serum creatinine, hemoglobin levels, and outcomes in critically ill COVID-19 patients requiring ICU admission.

## MATERIAL AND METHODS

### Study design and setting

A retrospective, single-center observational study evaluated all patients admitted to the Critical Care Unit (COVID-19 ICU) with moderate to severe COVID-19 disease at King Fahad University Hospital, Dammam, Eastern Province, Kingdom of Saudi Arabia, from January 2020 to August 2021. The study followed the ethical standards of the Helsinki Declaration and the institutional and national research committees. Ethical approval for the present study was received from the institutional review board at Imam Abdulrahman bin Faisal University. This study followed the Strengthening the Reporting of Observational Studies in Epidemiology (STROBE) guidelines.

### Inclusion Criteria

All patients aged ≥18 years with a positive SARS-CoV-2 real-time reverse transcription polymerase chain reaction (RT–PCR) test who required ICU admission and had a moderate to severe COVID-19 infection were included in the study.

### Exclusion Criteria

1. Age <18 years.
2. Pregnancy.
3. An incidental positive SARS-CoV-2 test without symptoms.
4. Mild disease and no requirement for ICU admission.
5. Patients with a diagnosis of chronic kidney failure.
6. End-stage renal disease on regular hemodialysis.
7. Acute kidney injury on admission, as defined by the RIFLE criteria [14].
8. Patients with evidence of gastrointestinal bleeding, melena, hematochezia, and vomiting of coffee-ground material on admission.

COVID-19 was diagnosed according to the Saudi Center for Disease Prevention and Control guidelines. Swabs were taken from each patient’s nasopharyngeal orifice/s and oropharynx. Sample investigations were performed by certified laboratory personnel following the manufacturer’s recommendations for the defined cutoff cycle threshold (CT) value for each target gene using RNA, ViiA7 RT–PCR (Thermo Fisher Scientific, Waltham, MA, USA), and Altona reagents (Altona Diagnostics, Hamburg, Germany) [15,16].

### Data collection

The data of the patients included were extracted from the Electronic Health Records system (QuadraMed). The following data were collected from eligible patients: 1) demographic data, clinical characteristics (sex, age, body mass index [BMI], and smoking status), and coexisting disease (hypertension, diabetes mellitus, obesity, cardiovascular diseases, chronic pulmonary diseases, and immunosuppressed status); 2) laboratory results (serum chloride levels, hemoglobin levels, creatinine, and inflammatory markers, namely, ferritin levels, CRP, LDH, and D-dimer), which were recorded on the first and third days of ICU admission; 3) the modality of oxygen support required (noninvasive and invasive mechanical ventilation), the duration of intubation and mechanical ventilation, a requirement for RRT, and length of ICU stay (ICU-LOS); and 4) mortality.

### Outcomes

The main objectives of this study were as follows: 1) to identify associations between hyperchloremia and inflammatory markers (CRP, ferritin, LDH, D-dimer), serum creatinine, and anemia; and 2) to investigate the impact of hyperchloremia on patient outcomes; modality of oxygen support (invasive vs. noninvasive mechanical ventilation), the duration of invasive ventilation, the number of patients requiring RRT, the length of ICU stay and mortality.

### Definitions

The following definitions were used to define the severity of COVID-19. Moderate COVID-19 disease: the presence of respiratory symptoms or radiographic findings (chest X-ray) of lower respiratory tract infection with oxygen saturation (SpO2) >94% without oxygen support. Severe disease: the presence of tachypnea (respiratory rate >30 breaths/minute) or SpO2 <94% with no oxygen support or chest X-ray showing >50% lung infiltration [17]. Serum chloride levels, CRP, ferritin, LDH, D-dimer, serum creatinine, and hemoglobin levels were noted on the first and third days of admission, and differences between respective values were recorded. Hyperchloremia was defined as a serum chloride level >106 mmol/L on the first or third day of admission or an increase in the serum chloride level >5 mmol/L over the first three days of admission [18,19]. Increases in serum ferritin, CRP, LDH, and creatinine levels between the first and third days of admission were considered at levels >95 ng/ml, >10 mg/L, >238 U/L, and >0.3 mg/dl, respectively [20]. A decrease in hemoglobin levels >0.5 g/dl from baseline between the first and third days of admission was considered significant [21].

### Statistical analysis

The data analysis was performed using the IBM Statistical Package for the Social Sciences (SPSS Statistics 26). Categorical variables were presented as frequencies and percentages, while continuous variables were presented as the median and interquartile range (IQR) if the data followed a normal distribution and as mean (SD) for symmetrical data. To assess the associations between the variables, we used the chi-square or Fisher exact probability test, and to compare the medians, we used the Mann–Whitney U test or Student’s t-test. Odds ratios (ORs) and 95% confidence intervals (CIs) were determined by multivariate analysis. Significance was considered at a p-value<0.05 with a CI set at 95%.

## RESULTS

A retrospective review was conducted during the study period on 420 patients admitted to the ICU with moderate to severe COVID-19 infection. After applying the exclusion criteria, 255 patients were included in the study. The demographic and clinical characteristics of the patients are presented in Table 1. Among the patients, 170 (66.7%) were males, and 85 (33.3%) were females. Most patients (108, 42.35%) were aged between 41 and 60 years; 93 (36.47%) patients were aged 61–80 years, 40 (15.69%) patients were younger than 40 years of age, and 14 (5.49%) patients were older than 80 years. The mean age was 56.8±15 years. A total of 36 (14.1%) patients were smokers. The predominant comorbidities identified were diabetes mellitus (52.2%), obesity (49%), hypertension (46.7%), cardiovascular disease (14.1%), and chronic lung disease (10.6%).

**Table 1.** Participant demographics

		Frequency	Percent
**Age (years)** **Mean±SD=56.8±15**	≤40	40	15.69
	41–60	108	42.35
	61–80	93	36.47
	>80	14	5.49
**Gender**	Male	170	66.7
	Female	85	33.3
**Smoking**	Yes	36	14.1
	No	219	85.9
**Comorbidities**	Obesity	125	49
	DM	133	52.2
	HTN	119	46.7
	CVS	36	14.1
	CLD	27	10.6
	Immunosuppression	6	2.4

Data are presented as frequencies (%), Mean (standard deviation). COVID-19 – Coronavirus Disease 2019; ICU – intensive care unit; DM – diabetes mellitus; HTN – hypertension; CVS – cardiovascular disease; CLD – chronic lung disease.

**Figure 1.**
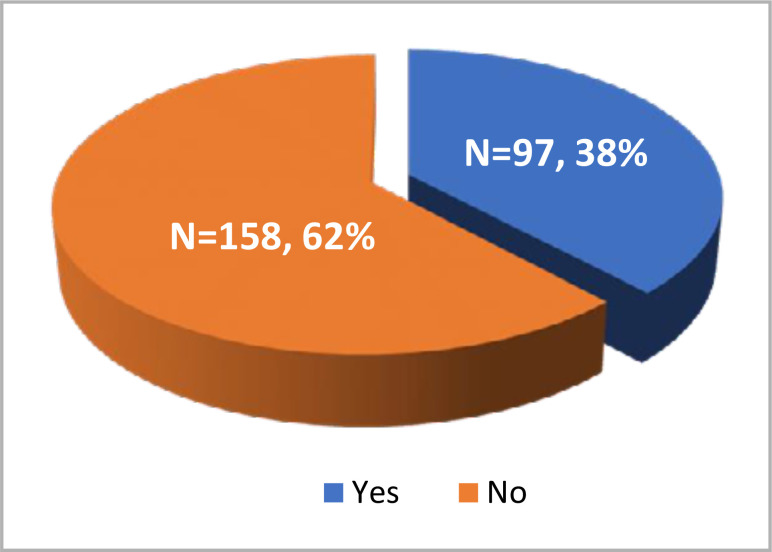
Hyperchloremia in critically ill COVID-19 patients (n=255)

Of the 255 COVID-19 patients included in the study, hyperchloremia (serum chloride >106 mmol/L) was observed in 97 (38%) patients either on the first or third day of ICU admission or with an increase in chloride levels >5 mmol/L during the first three days (Figure 1).

53 (54.6%) hyperchloremic patients had increased ferritin levels (>95 ng/ml), 6 (6.2%) had increased CRP levels (>10 mg/L), and 15 (15.5%) had increased LDH levels (>238 U/L) between the first and third days, while the numbers of non-hyperchloremic patients with increases in ferritin, CRP and LDH were 69 (43.7%), 10 (6.3%), and 9 (5.7%), respectively. Among the hyperchloremic patients, 45 (46.4%) patients had a decrease in hemoglobin >0.5 g/dL compared to 57 (36.1%) patients with normal serum chloride levels. Hyperchloremia was not significantly associated with increased ferritin, CRP, and hemoglobin levels in patients suffering from severe COVID-19. However, hyperchloremia was substantially correlated with increases in LDH and creatinine levels (p=0.01 and <0.0001, respectively) compared to normal serum chloride levels (Table 2).

**Table 2.** Association between hyperchloremia and elevated inflammatory markers, serum creatinine, hemoglobin decrease, and D-dimer levels (N=255)

	Hyperchloremia		P-values
	Yes	No	
**CRP increase, n (%)**	Yes	6 (6.2%)	10 (6.3%)	0.96
	No	91 (93.8%)	148 (93.7%)	
**Ferritin increase, n (%)**	Yes	53 (54.6%)	69 (43.7%)	0.089
	No	44 (45.4%)	89 (56.3%)	
**LDH increase, n (%)**	Yes	15 (15.5%)	9 (5.7%)	0.01
	No	82 (84.5%)	149 (94.3%)	
**Serum Creatinine increase, n (%)**	Yes	24 (24.7%)	13 (8.2%)	<0.0001
	No	73 (75.3%)	145 (91.8%)	
**Hb decrease n (%)**	Yes	45 (46.4%)	57 (36.1%)	0.103
	No	52 (53.6%)	101 (63.9%)	
**D Dimmer, n (%)**	Yes	62 (63.9%)	83 (52.5%)	0.075
	No	35 (36.1%)	75 (47.5%)	

CRP – C-reactive protein; LDH – lactate dehydrogenase; Hb – hemoglobin.

Requirements for intubation and mechanical ventilation were significantly higher among hyperchloremic patients. Sixty-six (68%) patients required invasive ventilation in the hyperchloremic group compared to seventy-six (48.1%) patients without hyperchloremia (p=0.003). Moreover, the mortality rate was higher among hyperchloremic patients. Fifty-five (56.7%) patients died in the hyperchloremic cohort compared to forty-nine (31%) patients without hyperchloremia (p=<0.0001). The number of patients who required continuous renal replacement therapy (CRRT), noninvasive ventilation, and ICU LOS was similar between groups. The associations between hyperchloremia and outcomes of interest are demonstrated in Table 3.

**Table 3.** Relationship between hyperchloremia and outcome measures

		Hyperchloremia		P-values
	Yes	No	
**Mortality, n (%)**	Yes	55 (56.7%)	49 (31%)	<0.0001
	No	42 (43.3%)	108 (68.4%)	
Dialysis (CRRT)*, n (%)	Yes	26 (26.8%)	29 (18.4%)	0.11
	No	70 (72.2%)	128 (81%)	
**Invasive mechanical ventilation, n (%)**	Yes	66 (68%)	76 (48.1%)	0.003
	No	31 (32%)	80 (50.6%)	
**Non-invasive ventilation, n (%)**	Yes	78 (80.4%)	139 (88%)	0.1
	No	19 (19.6%)	19 (12%)	
**ICU stay**	Median (IQR)	13 (7.5–21.5)	18 (9.5–22.8)	0.309
**Days to death**		17 (9.5–24)	19 (10.25–25)	0.36

Data are presented as frequencies (%), Median (interquartile range). *CRRT – continuous renal replacement therapy.

A multivariate analysis of the associations between hyperchloremia and outcomes, age, sex, and increases in inflammatory markers showed that hyperchloremia was substantially correlated with an increase in LDH (OR 3.028; p= 0.012), an increase in serum creatinine (OR 2.55; p=0.032), invasive mechanical ventilation (OR 2.2, p=0.003) and mortality (OR 2.392, p = 0.032). However, serum ferritin, CRP, D-dimer, and hemoglobin were not significantly correlated with hyperchloremia. Moreover, despite the significant increase in serum creatinine levels with hyperchloremia, the number of patients who required CRRT was not significantly associated with hyperchloremia. The multivariate analysis is presented in Table 4.

**Table 4.** Multivariate analysis of associations between hyperchloremia, outcomes, age, gender, and increase in inflammatory markers (N=255)

	Odds Ratios	95% Confidence Interval	P-values
**Gender**	1.012	(0.56–1.84)	0.97
**Age**	1.011	(0.98–1.04)	0.424
**CRP increase**	0.538	(0.17–1.76)	0.305
**Ferritin increase**	1.28	(0.69–2.39)	0.438
**LDH increase**	3.028	(1.3–7.2)	0.012
**Creatinine increase**	2.55	(1.09–5.98)	0.032
**Hb decrease**	1.299	(0.72–2.35)	0.385
**D-dimer increase**	0.927	(0.48–1.8)	0.822
**Invasive ventilation**	2.2	(1.3–3.8)	0.003
**Non-Invasive ventilation**	0.792	(0.35–1.78)	0.571
**Dialysis CRRT**	0.587	(0.26–1.34)	0.207
**Mortality**	2.392	(1.08–5.31)	0.032
**ICU stay**	0.95	(0.84–1.07)	0.411
**Days to death**	1.048	(0.94–1.17)	0.393

CRP – C-reactive protein; LDH – Lactate dehydrogenase; Hb – hemoglobin; CRRT – continuous renal replacement therapy.

## DISCUSSION

The incidence of hyperchloremia in the adult ICU has been previously reported to be between 20 and 40% [22,23]. Hyperchloremia, whether caused by the sepsis process or as a side effect of fluid resuscitation, appears to have a negative impact on patient outcomes. Hyperchloremia or increased chloride levels >5 mmol/L can lead to metabolic acidosis, AKI, anemia, and hemodynamic instability [24]. Unfortunately, data on the incidence of hyperchloremia in critically ill COVID-19 patients are limited. In this study, 38% of critically ill COVID-19 patients had hyperchloremia. A recent similar study by Amara et al. showed that the incidence of hyperchloremia in the COVID-19 ICU reached 21% [25].

The increased inflammatory response to COVID-19 has been identified as a major cause of morbidity and mortality. Moreover, elevated concentrations of proinflammatory markers such as IL-6, IL-18, and inflammatory chemokines have been associated with poor disease outcomes [26]. The cytopathic effects of SARS-CoV-2 and the propagation of proinflammatory responses can result in endothelial dysfunction and oxidative stress [27]. Oxidative stress is characterized by increased production of reactive oxygen species and low activity of antioxidants, resulting in cellular damage [28]. Viral infection can be associated with oxygen-free radical production secondary to mitochondrial dysfunction. Furthermore, the progression of oxidative stress due to an intense inflammatory response can cause impairment in the synthesis and release of nitric oxide from endothelial cells, resulting in endothelial dysfunction [29]. Previous studies have demonstrated that utilizing antioxidants during severe COVID-19 might help reduce oxidative stress and endothelial dysfunction and prevent inflammation-mediated tissue injury [30].

**Figure 2.**
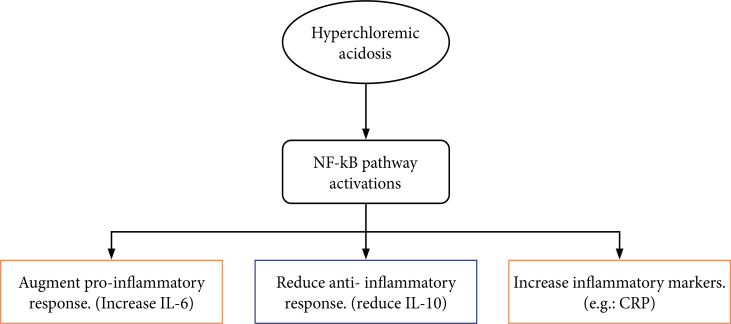
Effects of hyperchloremia on inflammatory response. NF-kB – nuclear factor kappa B; CRP – C-reactive protein

Chloride is an often-overlooked electrolyte but has a substantial impact on patient outcomes, as it can lead to disturbances in body physiology and hemostasis, including acid-base balance and immunomodulation disturbances [5]. Hyperchloremic acidosis can significantly augment proinflammatory mediators (IL-6) through the activation of the NF-kB pathway [7]. Activation of this pathway can result in increased CRP levels, which has a significant impact on the severity of COVID-19 [8]. Therefore, the present study explored the association between hyperchloremia and the propagation of inflammatory markers in critically ill COVID-19 patients. (Figure 2). However, we found no significant association between hyperchloremia or an increase in serum chloride levels >5 mmol/L over the first three days of ICU admission and an increase in CRP levels or serum ferritin in the present study. Our result is consistent with a recent report by Amara et al., which showed no significant association between hyperchloremia and an increase in inflammatory markers [25]. However, in the present study, a significant association was identified between hyperchloremia and an increase in serum LDH levels from day one to day three of admission. The number of hyperchloremic patients with an increase in LDH (>238 U/L) between the first and third days of admission was 15 (15.5%) (p=0.01). The correlation of serum LDH levels with in-hospital mortality in critically ill COVID-19 patients has been evaluated in previous literature. Elevated serum LDH was found to be an independent risk factor for in-hospital mortality [31]. Moreover, elevated LDH levels were correlated with an increased risk of hypoxia and respiratory failure in critically ill COVID-19 patients [32]. Therefore, early detection and avoiding the infusion of chloride-rich fluids might help prevent LDH elevation and mitigate COVID-19 complications in critically ill patients.

Previous research has shown a link between hyperchloremia and an increased risk of developing AKI. The proposed pathophysiology of AKI caused by hyperchloremia includes dysregulated tubule-glomerular feedback, activated by chloride reaching the macula densa and causing afferent arteriole vasoconstriction, leading to cortical tissue ischemia. [33]. The present study found a significant association between hyperchloremia and the development of AKI, defined by an increase in serum creatinine levels >0.3 mg/dl over 72 hours from admission (OR 2.55; p=0.032). In accordance with our results, Yunos NM et al. reported a strong association between hyperchloremia from infusing chloride-rich fluids and the risk of developing AKI [34]. In contrast to our findings, recent studies found no relationship between hyperchloremia and AKI in septic shock patients [35]. However, in this study, a requirement for RRT was nonsignificant among hyperchloremic patients compared to other patients, which is consistent with a recent study performed by Suetrong B et al. showing no significant association between hyperchloremia and a requirement for RRT in critically ill patients [36].

Several hematological complications have been reported during the COVID-19 pandemic. Hemolytic anemia has been linked to COVID-19 disease, either directly, as a cytopathic effect of viral infections, or indirectly, due to autoantibody production, resulting in autoimmune hemolytic anemia [37]. Multiple mechanisms have been proposed to explain the direct effect of COVID-19 on erythrocytes. The interaction between SARS-CoV-2 and CD147 facilitates viral entry into erythrocytes, leading to dysregulation of CD147 and subsequently causing hemolysis [38]. Moreover, erythrocyte band 3 protein is primarily present in mature erythrocytes and is necessary for maintaining membrane integrity and oxygen release [39]. The interaction between the SARS-CoV-2 spike protein and band 3 can affect the oxygen transportation ability and cause hypoxia, subsequently increasing the risk of erythrocyte injury [40]. Autoimmune hemolytic anemia has been observed in up to 14.7% of critically ill COVID-19 patients [41]. A previous report demonstrated a link between autoimmune hemolysis and worse outcomes in COVID-19 patients [41]. The exact mechanism of autoimmune hemolytic anemia in COVID-19 remains unclear. However, the intense inflammatory response, dysregulation of the complement system, and the immune complex have been suggested as the causes of autoantibody production [42]. Increased blood viscosity is another hematological complication linked to COVID-19. Hyperviscosity syndrome (HVS) can result in devastating complications due to poor tissue perfusion, increased vascular resistance, thrombosis, and subsequent organ dysfunction. The exaggerated immune response and massive release of proinflammatory cytokines that cause hyperfibrinogenemia in COVID-19 is one possible mechanism for HVS development [43]. However, our study explored the association between hyperchloremia and a decrease in hemoglobin levels in critically ill COVID-19 patients. Notably, hyperchloremic acidosis can increase the risk of coagulopathy and subsequent anemia. Moreover, AKI associated with hyperchloremia might affect erythropoietin levels, resulting in anemia. In line with previous studies [25,44], no notable association was found between hyperchloremia and anemia in this study. Among the hyperchloremic patients, 45 (46.4%) showed a decrease in hemoglobin levels >0.5 g/dL compared to 57 (36.1%) patients with normal serum chloride levels.

The present study found a significant association between hyperchloremia and the need for invasive mechanical ventilation in critically ill COVID-19 patients. Sixty-six (68%) patients required intubation and mechanical ventilation in the hyperchloremic group compared to seventy-six (48.1%) patients without hyperchloremia (p=0.003). We did not find any reports from previous studies explaining the impact of hyperchloremia on lung function or the requirement for invasive mechanical ventilation. However, this may be attributed to several reasons. Firstly, elevated serum LDH levels associated with hyperchloremia can significantly affect lung function and lead to severe hypoxia [32]. Secondly, most critically ill patients are frequently exposed to a large volume of chloride-rich fluid during resuscitation, which can result in volume overload and affect lung mechanics. Lastly, hyperchloremic metabolic acidosis might be associated with an increased respiratory drive as a compensatory mechanism in spontaneously breathing patients, which might increase the risk of self-inflicted lung injury [45]. Although the effect of hyperchloremia on respiratory function appears to be a separate element, further research is necessary to determine whether a causal association exists between hyperchloremia and respiratory function.

Hyperchloremia has been identified as an independent risk factor for ICU mortality during the acute phase of critical illness. Moreover, positive fluctuation in serum chloride levels greater than 1 mmol/L in the first three days of ICU admission was correlated with increased 30-day mortality by 8% [46]. However, most studies examining the link between hyperchloremia and ICU mortality have yielded contradictory results. A retrospective observational study conducted by Neyra et al. demonstrated a strong association between hyperchloremia or worsening serum chloride levels in the first three days of ICU admission and hospital mortality [19]. Similarly, de Vasconcellos et al. found that hyperchloremia or increased serum chloride levels within 48 hours of ICU admission were significantly correlated with adverse outcomes and in-hospital mortality [47]. In contrast, a post hoc analysis of the HYPER2S trial performed by Morgane et al. did not find any correlation between hyperchloremia and in-hospital deaths [48]. However, in the current study, the mortality rate was higher among patients with hyperchloremia or increased serum chloride levels >5 mmol/L within 72 hours of ICU admission. Fifty-five (56.7%) patients died in the hyperchloremic cohort compared to forty-nine (31%) patients without hyperchloremia (p<0.0001). However, the effects of hyperchloremia on patient outcomes may be minor when weighed against the inherent risks of acute illness. More research is needed to determine the impact of hyperchloremia on the outcomes of critically ill COVID-19 patients.

The strength of this study is that we explored the impact of hyperchloremia in a specific group of ICU patients who were positive for COVID-19, whereas previous studies demonstrated the effects of hyperchloremia in most ICU patients.

The present study has several limitations that must be taken into consideration. Firstly, the study was conducted at a single center with a relatively small sample size, which may limit the generalizability of the results to other populations. As a result, large-scale multicenter studies are advised. Furthermore, because the study was conducted retrospectively, the link between hyperchloremia and patient outcomes is difficult to confirm. Future prospective studies are therefore essential to better understand the effects of hyperchloremia on COVID-19 patients.

## CONCLUSION

Hyperchloremia (Cl >106 mmol/L) or an increased serum chloride level >5 mmol/L in the first three days of ICU admission was significantly associated with a negative impact on patient outcomes, including the development of AKI, a requirement for invasive mechanical ventilation, and mortality in critically ill COVID-19 patients. However, hyperchloremia-induced AKI was not associated with an increased requirement for RRT. Hyperchloremia or an increased serum chloride level > 5 mmol/L was not significantly associated with increased ferritin levels, CRP, and D-dimer. Furthermore, we found a significant correlation between serum LDH and hyperchloremia or a positive fluctuation of serum chloride levels greater than 5 mmol/L within the first three days of ICU admission. Ultimately, a larger prospective study is required to determine the impact of hyperchloremia on the outcomes of critically ill COVID-19 patients.
